# Supplementary material for: Iterative improvement in the automatic modular design of robot swarms
Source: PeerJ Comput Sci. 2020 Dec 7;6:e322. doi: 10.7717/peerj-cs.322 (PMC7924708; doi:10.7717/peerj-cs.322)
Supplement: Supplemental Information 3 [file peerj-cs-06-322-s003.zip › argos3/doc/api/standalone/a00600_source.html]

ARGoS: plugins/simulator/physics\_engines/dynamics2d/dynamics2d\_box\_model.cpp Source File


- Main Page
- Related Pages
- Namespaces
- Classes
- Files

- File List
- File Members

# plugins/simulator/physics\_engines/dynamics2d/dynamics2d\_box\_model.cpp

Go to the documentation of this file.

```
00001 
00007 #include "dynamics2d_box_model.h"
00008 #include "dynamics2d_gripping.h"
00009 #include "dynamics2d_engine.h"
00010 
00011 namespace argos {
00012 
00013    /****************************************/
00014    /****************************************/
00015 
00016    CDynamics2DBoxModel::CDynamics2DBoxModel(CDynamics2DEngine& c_engine,
00017                                             CBoxEntity& c_entity) :
00018       CDynamics2DStretchableObjectModel(c_engine, c_entity) {
00019       /* Get the size of the entity */
00020       CVector3 cHalfSize = c_entity.GetSize() * 0.5f;
00021       /* Create a polygonal object in the physics space */
00022       /* Start defining the vertices
00023          NOTE: points must be defined in a clockwise winding
00024       */
00025       cpVect tVertices[] = {
00026          cpv(-cHalfSize.GetX(), -cHalfSize.GetY()),
00027          cpv(-cHalfSize.GetX(),  cHalfSize.GetY()),
00028          cpv( cHalfSize.GetX(),  cHalfSize.GetY()),
00029          cpv( cHalfSize.GetX(), -cHalfSize.GetY())
00030       };
00031       const CVector3& cPosition = GetEmbodiedEntity().GetOriginAnchor().Position;
00032       CRadians cXAngle, cYAngle, cZAngle;
00033       GetEmbodiedEntity().GetOriginAnchor().Orientation.ToEulerAngles(cZAngle, cYAngle, cXAngle);
00034       /*
00035        * Create body and shapes
00036        */
00037       cpBody* ptBody;
00038       if(GetEmbodiedEntity().IsMovable()) {
00039          /* The box is movable */
00040          SetMass(c_entity.GetMass());
00041          /* Create the body */
00042          ptBody =
00043             cpSpaceAddBody(GetDynamics2DEngine().GetPhysicsSpace(),
00044                            cpBodyNew(GetMass(),
00045                                      cpMomentForPoly(GetMass(),
00046                                                      4,
00047                                                      tVertices,
00048                                                      cpvzero)));
00049          ptBody->p = cpv(cPosition.GetX(), cPosition.GetY());
00050          cpBodySetAngle(ptBody, cZAngle.GetValue());
00051          /* Create the shape */
00052          cpShape* ptShape =
00053             cpSpaceAddShape(GetDynamics2DEngine().GetPhysicsSpace(),
00054                             cpPolyShapeNew(ptBody,
00055                                            4,
00056                                            tVertices,
00057                                            cpvzero));
00058          ptShape->e = 0.0; // no elasticity
00059          ptShape->u = 0.7; // lots contact friction to help pushing
00060          /* The shape is grippable */
00061          SetGrippable(new CDynamics2DGrippable(GetEmbodiedEntity(),
00062                                                ptShape));
00063          /* Set the body so that the default methods work as expected */
00064          SetBody(ptBody, c_entity.GetSize().GetZ());
00065          /* Friction with ground */
00066          SetLinearFriction(0.0f, 1.49f);
00067          SetAngularFriction(0.0f, 1.49f);
00068       }
00069       else {
00070          /* The box is not movable */
00071          /* Create a static body */
00072          ptBody = cpBodyNewStatic();
00073          ptBody->p = cpv(cPosition.GetX(), cPosition.GetY());
00074          cpBodySetAngle(ptBody, cZAngle.GetValue());
00075          /* Create the shape */
00076          cpShape* ptShape =
00077             cpSpaceAddShape(GetDynamics2DEngine().GetPhysicsSpace(),
00078                             cpPolyShapeNew(ptBody,
00079                                            4,
00080                                            tVertices,
00081                                            cpvzero));
00082          ptShape->e = 0.0; // No elasticity
00083          ptShape->u = 0.1; // Little contact friction to help sliding away
00084          /* This shape is normal (not grippable, not gripper) */
00085          ptShape->collision_type = CDynamics2DEngine::SHAPE_NORMAL;
00086          /* Set the body so that the default methods work as expected */
00087          SetBody(ptBody, c_entity.GetSize().GetZ());
00088       }
00089    }
00090    
00091    /****************************************/
00092    /****************************************/
00093 
00094    REGISTER_STANDARD_DYNAMICS2D_OPERATIONS_ON_ENTITY(CBoxEntity, CDynamics2DBoxModel);
00095 
00096    /****************************************/
00097    /****************************************/
00098 
00099 }
```

---

Generated on 10 Jul 2018 for ARGoS by 
 1.6.1
